# Hyperpolarized NMR Reveals Low-Populated Folding Intermediates in DNA

**DOI:** 10.1021/jacs.5c17542

**Published:** 2025-12-08

**Authors:** Milan Zachrdla, Ertan Turhan, Michala Bučková, Robert Hänsel-Hertsch, Lukáš Trantírek, Dennis Kurzbach

**Affiliations:** † Institute of Biological Chemistry, Faculty of Chemistry, 27258 University of Vienna, Währinger Str. 38, 1090 Vienna, Austria; ‡ Central European Institute of Technology, 37748 Masaryk University, 625 00 Brno, Czech Republic; § National Centre for Biomolecular Research, Faculty of Science, Masaryk University, Kamenice 5, 625 00 Brno, Czech Republic; ∥ Center for Molecular Medicine Cologne (CMMC), Faculty of Medicine and University Hospital Cologne, 14309 University of Cologne, 50931 Cologne, Germany; ⊥ Department of Translational Genomics, Faculty of Medicine and University Hospital Cologne, 14309 University of Cologne, 50931 Cologne, Germany; ¶ Cologne Excellence Cluster on Cellular Stress Responses in Aging-Associated Diseases (CECAD), University of Cologne and University Hospital Cologne, 50931 Cologne, Germany; □ Institute of Human Genetics, University Hospital Cologne, 50931 Cologne, Germany

## Abstract

Nuclear magnetic resonance (NMR) spectroscopy is the only biophysical technique capable of characterizing nucleic acid structures at atomic resolution under near-physiological liquid-state conditions. Still, it is fundamentally limited by intrinsically low sensitivity, particularly when analyzing high-molecular-weight, low-abundance, or polymorphic targets, such as DNAs (DNA). In this study, we demonstrate that hyperpolarized aqueous buffers generated via dissolution dynamic nuclear polarization (dDNP) significantly enhance the ^1^H NMR signals of multiple DNA motifs. The resonances of labile imino and amino protons of DNAs dissolved in hyperpolarized buffers are enhanced up to ∼200-fold and ∼370-fold, respectively. These intense signals serve a 2-fold purpose: (i) as structural fingerprints of DNA folding topologies and (ii) they enable the direct observation of low-populated folding intermediates in DNA polymorphs, such as G-quadruplexes (G4) and i-motifs (iM), which remain undetectable by standard methods. Thus, our findings establish hyperpolarized NMR as a high-sensitivity method for probing DNA structures and folding intermediates across a wide range of motifs, opening possible avenues in liquid biopsy applications and cell-free DNA.

## Graphical abstract

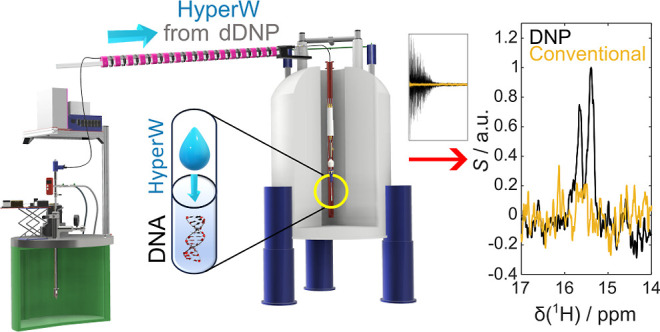

## Introduction

Nuclear magnetic resonance (NMR) spectroscopy remains unparalleled in its ability to resolve the structural and dynamic landscapes of biomolecules in their native solution state.^1–3^ This capability makes NMR a central tool in chemical and biological sciences. However, despite its broad application, the technique suffers from a fundamental limitation: its inherently low sensitivity.^4,5^ As a result, conventional biomolecular NMR typically demands analyte concentrations that far exceed physiological conditions.^6^ This discrepancy severely limits the scope of NMR-based studies, particularly for low-concentration samples or transiently formed structures.^7–9^

In recent years, hyperpolarization by dissolution dynamic nuclear polarization (dDNP) has reemerged as a powerful strategy to address this sensitivity bottleneck.^7,8^ By hyperpolarizing NMR-active nuclei *ex situ* at cryogenic temperatures and subsequently dissolving the polarized sample and transferring it rapidly to an NMR spectrometer for detection, dDNP enables signal enhancements of several orders of magnitude.^9–15^ Among the various implementations of this approach, the use of hyperpolarized water (HyperW) has garnered particular attention.^16–32^ Within this approach, water acts as the polarization reservoir, transferring its enhanced proton polarization to labile hydrogen sites within biomolecules via proton exchange and nuclear Overhauser mechanisms. The advantage of using HyperW as a sensitivity vector is 2-fold. First, the DNP process is independent of the target molecule. Hence, the latter does not experience harsh conditions during sample dissolution and transfer, rendering dDNP applicable also to molecules with secondary and tertiary structures based on noncovalent interactions. Second, the hyperpolarization reservoir is relatively long-lived as water displays much slower nuclear relaxation than typical biomacromolecules.^16–32^ Thus, the acquisition window for hyperpolarized detection is prolonged.

HyperW has been successfully applied to peptides, as well as to intrinsically disordered and folded proteins, providing signal enhancements of up to >700 times^33^ over thermal equilibrium conditions. This enhancement enabled previously inaccessible NMR applications, including the characterization of protein structures at micromolar concentrations, the acquisition of novel insights into real-time interactions and folding, and the study of proteins in a membrane-bound context.^2,12,16–30,32,33^ A notable feature of the HyperW application, which emerged from the protein-based studies, is that the hyperpolarization transfer from solvent to target, i.e., the NMR signal strength, critically depends on proton exchange: only protein residues efficiently interacting with water can benefit from HyperW, effectively masking sequestered sites.^30^

As a result, DNAs have not yet been investigated using dDNP/HyperW. This methodological gap has not been coincidental but is rooted in the specific proton exchange regime associated with base-pair opening in many canonical and noncanonical DNA motifs: The anticipated slow proton exchange kinetics rendered such attempts unpopular. However, Schwalbe, Frydman, and co-workers recently reported the successful application of HyperW to the guanine-sensing riboswitch aptamer domain of the xpt-pbuX operon from *Bacillus subtilis*, observing that HyperW-induced enhancements extended to all G and U imino protons involved in canonical G–C and A–U Watson–Crick (W–C), as well as noncanonical W–C-type G–U base pairs stabilizing the RNA aptamer structure.^28^ These results suggested that for double-stranded (B-form) DNA, which generally exhibits lower thermodynamic stability and thus increased base pair opening dynamics compared to RNA, HyperW could deliver at least comparable signal amplification.

With this in mind, we set out to evaluate the potential of dDNP and HyperW for structural studies not only of DNA structures stabilized by Watson–Crick G-C and A-T base pairs, but also of a variety of other DNA motifs stabilized by Hoogsteen G–G, A–T, CH^+^.G, and noncanonical W–C-type C.CH^+^ and G–T base pairs. These include commonly studied forms such as DNA triplexes and tetra-stranded structures, represented by G-quadruplexes, i-motifs, and their hybrids.

We demonstrate that hyperpolarized water can serve as a powerful sensitivity enhancer for imino and amino protons in DNA segments stabilized by various canonical and noncanonical base pairs, delivering up to a 370-fold increase in ^1^H signal compared to its thermal equilibrium reference, provided that key experimental parameters, particularly NMR detection pulse timing, are optimized. Notably, we observed that the signal enhancements were not solely dictated by the inherent stabilities of individual base pairs (G–C > A–T) but rather scaled with the expected base-pair opening/closing rates: we demonstrate that this scaling enables the direct observation of low-populated folding intermediates species along the kinetic-partitioning-based folding trajectory of DNA polymorphs, such as G-quadruplexes (G4) and i-motifs (iM).

We thus find that HyperW is not only a tool to enhance the S/N ratio of NMR spectra but can also provide information that is otherwise inaccessible by conventional NMR techniques.

## Results and Discussion

We first implemented a workflow that closely follows the established methodology of earlier NMR applications with hyperpolarized water (Figure 1).^2,30,32,34^ In our experiments, H_2_O (supplemented with 30% v/v glycerol-d_8_ and 15 mM TEMPOL as polarization agent) was hyperpolarized at cryogenic conditions (*B*_0,DNP_ = 6.7 T, *T*_DNP_ = 1.4 K, ν_μw_ = 188.0 GHz). After a 2 h polarization build-up, the frozen water pellet was rapidly dissolved using a preheated deuterated buffer (15 bar/220 °C) and transported to the NMR spectrometer while maintaining a constant magnetic field (*B*_0,transfer_ > 37 mT).^35,36^ At this point, we employed a Hybrid Sample Shuttling System (HySSS.v2),^37^ a prototype setup designed to ensure rapid and controlled mixing of the HyperW with an aqueous DNA solution directly within an NMR tube. The resulting mixture was then (2.5 s after the moment of dissolution) analyzed using a series of one-dimensional ^1^H NMR spectra (Figure 1).

**Figure 1:**
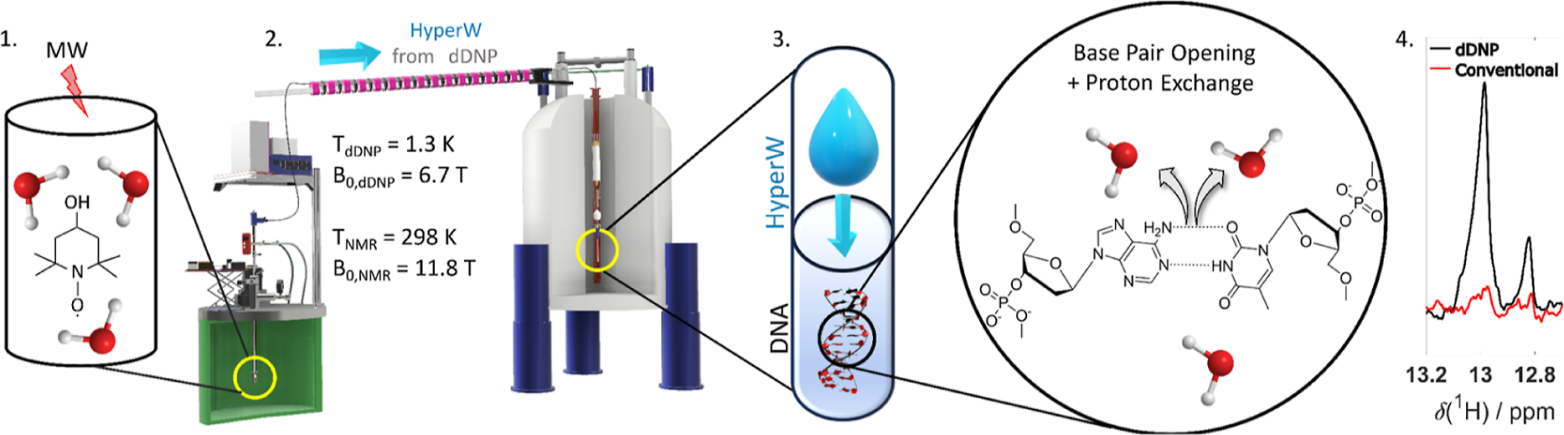
Proposed workflow for hyperpolarized DNA. First, water is hyperpolarized by DNP using cryogenic dynamic nuclear polarization. Then, upon rapid dissolution, the hyperpolarized water is transferred to the NMR spectrometer for detection. The hyperpolarized solvent is then mixed *in situ* within the NMR spectrometer with a DNA target. Finally, fast spectra acquisition with boosted ^1^H-sensitivity can then be recorded for a variety of DNA motifs.

One major challenge was the strong polarization of the water protons (centered around ∼ 4.7 ppm), which led to dominant solvent resonance signals if excitation was not selective.^30^ To leave the water resonance unaffected by the excitation scheme, we employed selective ^1^H pulses (Gauss.1000) that excited only the spectral region between 9 and 16 ppm (see Experimental Section). While the recovery delay between consecutive excitations of ≤0.3 s was employed earlier to access labile solvent-exposed sites in RNA and proteins,^28,38^ we found that recovery delays >0.5 s were often essential to allow water hyperpolarization to exchange efficiently with the imino sites in DNA; nuclear overhauser spectroscopy (NOESY) data demonstrating the slow water-imino proton exchange are provided in the Supporting Information.

In the following, we will report hyperpolarized spectra of six different DNA motifs (cf. Table 1). Each data set is reported together with two reference spectra:

a The thermal equilibrium reference spectrum (denoted TE) of the sample used for dDNP with its highly deuterated buffers (2% H_2_O, 98% D_2_O), but after decay of the hyperpolarization.
b The conventional NMR spectrum recorded in NMR-optimized (90% H_2_O/10% D_2_O) buffers (denoted REF_H2O_).

**Table 1:** List of DNA Constructs Employed in the Present Study

name	structural motif	sequence (5′ → 3′)	refs
DDD	B-DNA	(CGCGAATTCGCG).(CGCGAATTCGCG)	^41^
iTRP	Triplex/H-DNA	GAGAGAACCCCTTCTCTCTTTCTCTCTT	^48^
c-myc-G4	G-quadruplex	TGAGGGTGGGTAGGGTGGGTAA	^49^
TBA-Gt	G-triplex	GGTTGGTGTGG	^56^
T121–6-iM	i-motif	CCCCCCTCCCCCCTTCCCCCCTCCCCCC	^59^
LL3	G-quadruplex/i-motif hybrid	TCGTTCCGTTTTTCGTTCCGT	^61^

This differentiation is essential, since the imino and amino signal strength in the absence of any hyperpolarization scales with the degree of solvent deuteration. Thus, while TE is used to calculate the typically reported enhancement factor ε, REF_H_2_O_ is used to compute a factor ε*,^20^ defined as the signal-to-noise ratio (SNR) obtained by HyperW divided by the SNR obtained with the NMR-optimized sample scaled by the square root of the respective measurement times. Hence, ε* reports on how much the SNR was improved compared to optimized conventional NMR spectra.

Note that in cases where no signals could be recorded with the TE sample despite extensive signal averaging, we report enhancement factors as the SNR of the hyperpolarized spectrum. This corresponds to the lower limit of the enhancement, i.e., ε > SNR. Hence, in such a case, the enhancement is estimated conservatively. Representative ε and ε* factors are listed in Table 2 (for the complete list, see the Supporting Information) and are shown in every figure along with the number of scans (ns) used to acquire TE and REF_H_2_O_ spectra.

**Table 2:** Imino Signal Enhancements ε and ε* Obtained for the Different DNA Constructs^a^

construct	ε	ε*
DDD	126.5 ± 7.5	6.4 ± 0.4
iTRP	201.5 ± 86.3	49.9 ± 13.5
c-myc-G4	55.6 ± 13.9	4.0 ± 1.0
TBA-Gt	33.8 ± 1.6	15.0 ± 3.1
T121–6-iM	14.6 ± 1.3	2.2 ± 0.2
LL3	95.7 ± 4.6	3.4 ± 0.2

a Values are reported for the strongest imino signal in the hyperpolarized spectrum. Errors were obtained from the uncertainty in signal intensities due to spectral noise when calculating the enhancement factors (see Experimental Section for details).

### HyperW Enhancement for DNA Depends on Base-Pair Opening Dynamics

For initial scouting experiments, we used the Dickerson-Drew dodecamer (DDD; Table 1); an established and extensively studied model for right-handed B-form duplexes stabilized by canonical Watson–Crick base pairs (Figure 2a).^39,40^

**Figure 2:**
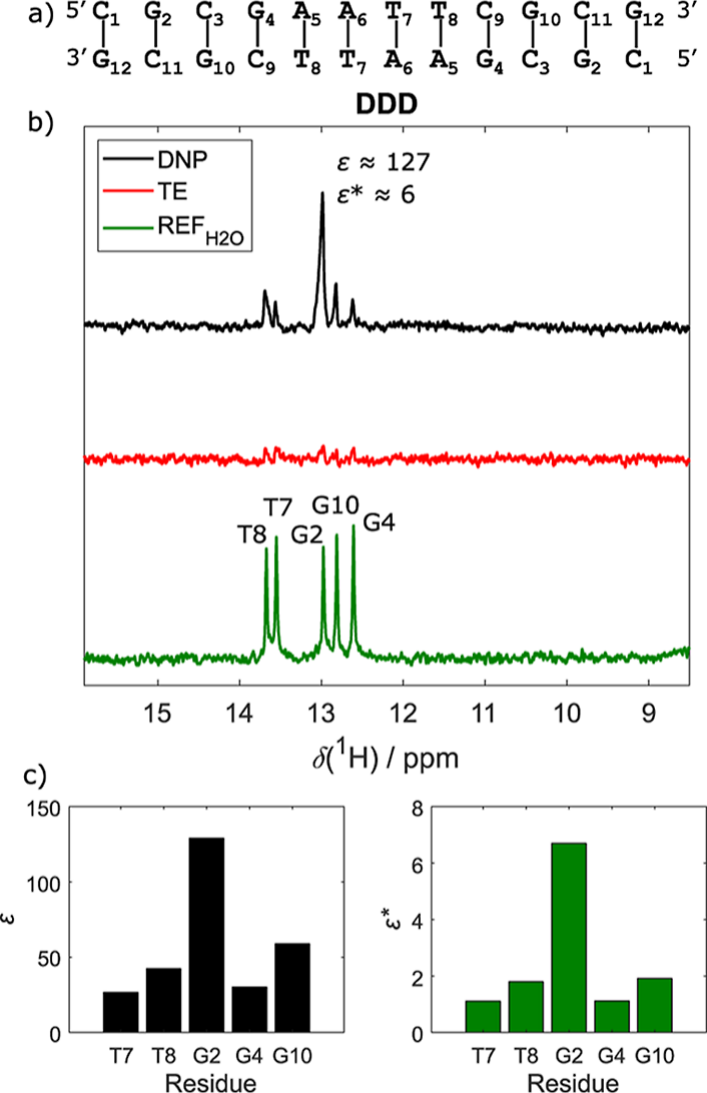
Dickerson-Drew dodecamer. (a) Schematic representation of DDD base-pairing. (b) Comparison of the hyperpolarized spectrum of DDD (black), thermal equilibrium (red), and standard NMR reference (green). Enhancements are indicated for the most intense peak. The hyperpolarized spectrum was recorded with 3 scans, the TE with 460 scans, and the REF_H_2_O_ with 64 scans. (c) Residue-specific enhancement factors ε and ε*. The resonance assignment was obtained from ref 41.

The imino region of the acquired hyperpolarized ^1^H spectrum (Figure 2b) revealed five distinct resonances correponding^60,61^ to guanines (G) and thymines (T) involved in Watson–Crick (W–C) A5–T8, A6–T7, G2–C11, C3–G10, and G4–C9 base pairs (bp). These resonances were also observed faintly in the TE spectrum. In line with previous studies^41–43^ reporting extensive terminal base pair freeing, hence, line broadening, the G12 imino proton was detected neither in the hyperpolarized nor in the thermal equilibrium spectrum .

The comparison of hyperpolarized and TE spectra revealed significant signal enhancements for all detected resonances (Figure 2b). However, variations in ε and ε* were observed between the different types of bp (G–C vs A–T). This was expected due to their inherent differences in stability. Less expectedly, we also found variations in ε between bp of the same type. While the signal intensity for G4-C9 was enhanced by ε ≈ 30, the imino proton from G2-C11 was enhanced by ε ≈ 130. Similarly, the signal for A5-T8 was enhanced to a larger extent (ε ≈ 40) compared to that of A6-T7 (ε ≈ 30).

These differences indicate that ε is not exclusively governed by the bp type and its specific stability (G–C > A–T) but further reflects the contribution from the position-specific frequency of base pair opening/closing, which is known to propagate from the fragment termini: bp opening is documented to be frequent for the first-2nd bp (counting from the 5′ end), moderate for the third-4th base pair, and negligible from the fifth base pair inward.^44–47^

ε* relative to REF_H_2_O_ scaled between 7 and 1, indicating that HyperW enhanced signal amplitudes by significantly above the SNR obtained with conventional methods, even despite extensive signal averaging (cf. Table 2 and Figure 2b). Notably, no signal broadening or changes in signal positions were observed for the DDD sample relative to the thermal equilibrium spectrum.

Next, to assess the generalizability of these observations, we acquired a hyperpolarized ^1^H NMR spectrum of an iTRP construct (Table 1 and Figure 3a), which folds into an intramolecular triplex under mildly acidic conditions.^48^ The triplex structure consists of a hairpin motif stabilized by W–C base pairing and a single-stranded 3′-end extension that folds back and binds into the major groove of the hairpin via Hoogsteen base pairing to form T·A–T and C^+^·G–C triplets.

**Figure 3:**
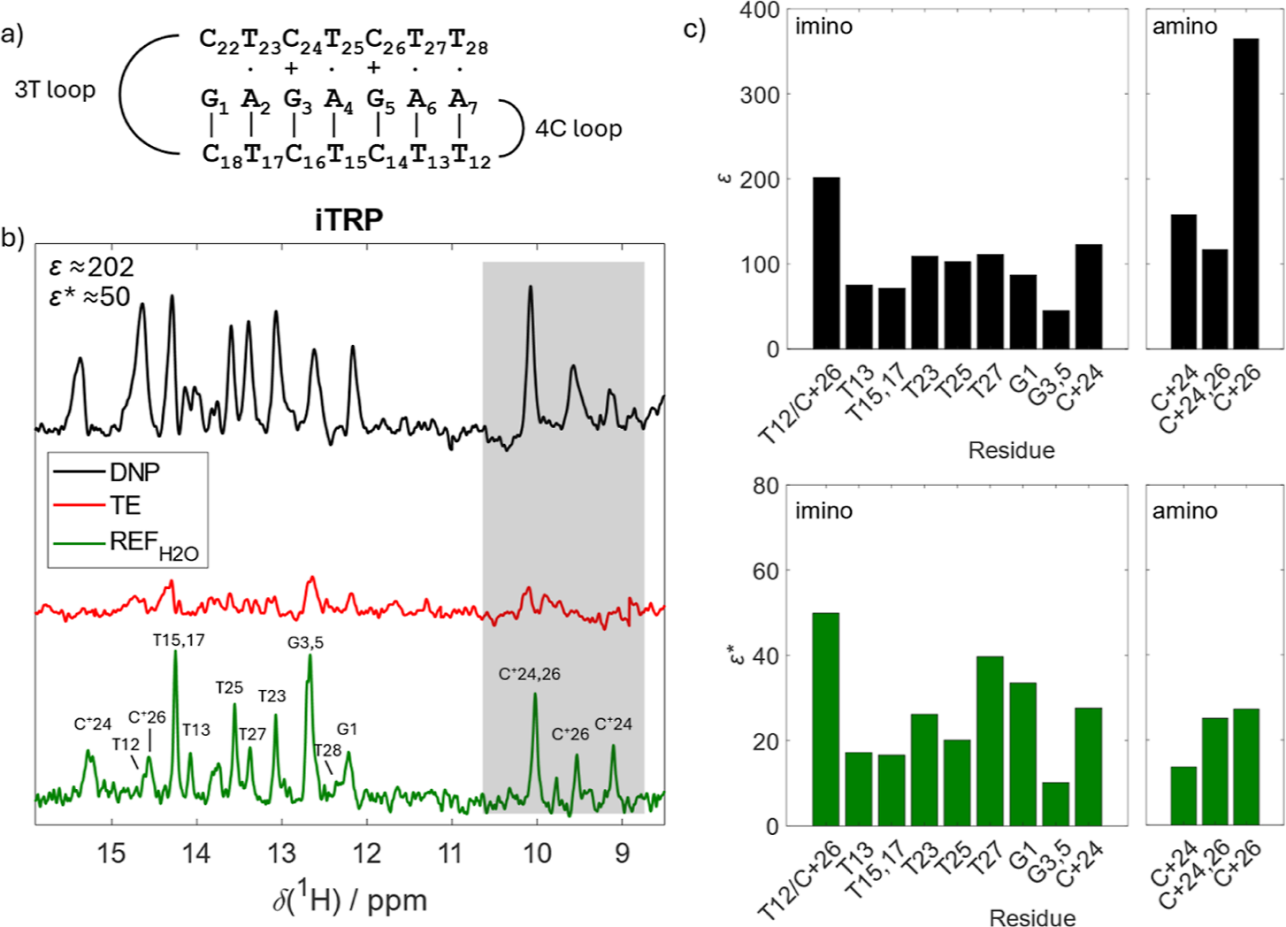
Triplex/H-DNA (iTRP). (a) Schematic representation of iTRP structure. (b) Comparison of the hyperpolarized spectrum of iTRP (black), thermal equilibrium (red), and standard NMR reference (green). Enhancements are indicated for the most intense peak. The hyperpolarized spectrum was recorded with 10 scans (within a single dDNP shot), the TE with 20,480 scans, and the REF_H_2_O_ with 2048 scans. (c) Residue-specific enhancement factors ε and ε*. Amino protons highlighted by the gray box in panel b are shown on the right-hand side. The resonance assignment was obtained from ref 48.

The comparison of the dDNP and REF_H_2_O_ spectra of iTRP revealed substantial enhancements for the majority of imino and amino proton signals, with ε up to 200 and 370, respectively, again without any significant line broadening. (Note: this observation will be crucial for the downstream analysis of G-quadruplex data.) Although the signal patterns were similar between the hyperpolarized and REF_H_2_O_ spectra (Figure 3b), both of which correspond to the previously reported spectrum of iTRP,^48^ shifts in the positions of some signals were observed, for example, for G1, T12, or C24^+^. These can be attributed to changes in buffer composition under dDNP conditions, particularly the varying degree of solvent deuteration. Indeed, the shifts were exclusively observed between dDNP and REF_H_2_O_ spectra, but not between dDNP and TE spectra (Figure 3b).

Notably, the terminus-to-center gradient of decreasing enhancement observed for DDD was also observed for iTRP in both Watson–Crick and Hoogsteen base-paired strands. The enhancements for imino protons involved in 5′ terminal W–C G1–C18 and A7–T12 bp in the hairpin were notably higher (∼3-fold) compared to those of the more centrally positioned G3/5–C16/14 and A6–T13 bp (Figure 3c). The observed relative differences in ε were consistent not only with the expected levels of terminal base pair opening frequencies, but also with the previously reported pronounced chemical exchange for T12 in the T28·A7–T12 triplet of iTRP.^48^ Similarly, the ε* for the Hoogsteen-paired T27, located near the triplex 3′-terminus, was ∼2-fold higher than that of the more centrally positioned Hoogsteen-paired T25 (Figure 3c). In this regard, the comparatively lower ε* values observed for T17 and T23 (both part of the identical triplet; T23·A2–T17) suggests reduced dynamics, most likely constrained by the 3T loop segment.

Collectively, the DDD and iTRP data demonstrate the efficacy of the HyperW approach in enhancing imino (and amino) ^1^H NMR signals originating from both canonical (W–C) and noncanonical (Hoogsteen) base pairs. The observation of larger enhancements for imino protons in structurally dynamic, rather than conformationally constrained, environments suggests a correlation between signal enhancement and base pair dynamics, as well as opening frequencies.

### Hyperpolarized NMR Enables Detection of DNA Folding Intermediates

To further validate and extend the HyperW approach to noncanonical DNA structures, we acquired hyperpolarized ^1^H NMR data on the c-myc-G4, TBA-Gt, T121–6-iM, and LL3 constructs (Table 1).

#### c-myc-G4

The c-myc-G4 sequence corresponds to a variant of the c-myc promoter gene, which folds into a compact, stable G-quadruplex structure stabilized by three stacked G-tetrads. Each is held together by four G.G Hoogsteen base pairs (Table 1; Figure 4a).^49^ The comparison of the hyperpolarized and TE spectra (Figure 4b) for G4 revealed two features:

i The HyperW enhanced resolved signals involved in Hoogsteen G.G base pairs—ε and ε* factors up to 56 and 4, respectively.
ii The hyperpolarized spectrum displayed signals that were significantly broadened and not present in the TE or REF_H_2_O_ counterparts (Figure 4b).

**Figure 4:**
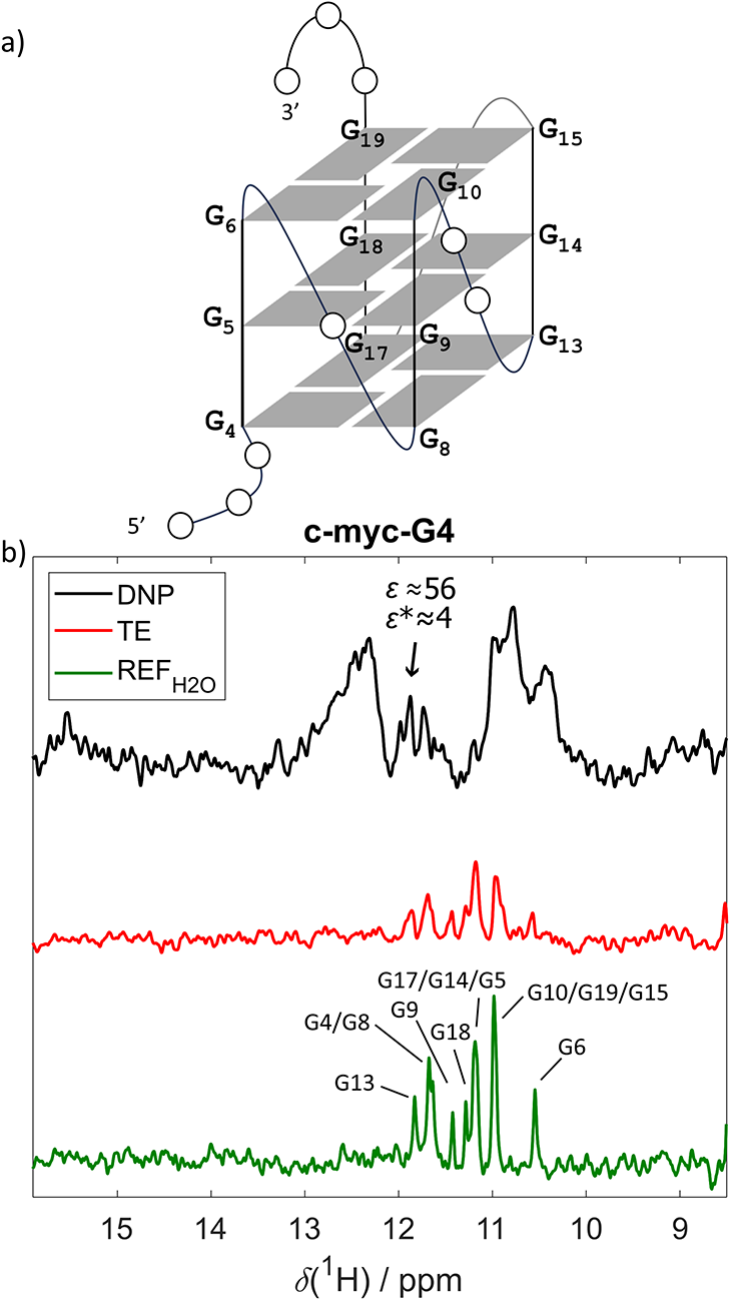
G-quadruplex (c-myc-G4). (a) Schematic representation of c-myc-G4 structure. (b) Comparison of the hyperpolarized spectrum of c-myc-G4 (black), thermal equilibrium (red), and standard NMR reference (green). Enhancements are indicated for G13. The hyperpolarized spectrum was recorded with 3 scans, the TE with 2048 scans, and the REF_H_2_O_ with 64 scans. The resonance assignment was obtained from ref 49.

The appearance of broad, unresolved peaks centered at 10.4, 10.8, and 12.4 ppm (Figure 4b), specific to the dDNP spectrum is indicative of the presence of multiple dynamic species coexisting alongside the G-quadruplex, leading to overlapping and/or exchange-broadened signals. (Note: the absence of any line broadening in the DDD and iTRP scouting experiments, as well as in G-quadruplex signals in the region between 11 and 12 ppm, rules out signal broadening due to the dDNP-typical transfer and mixing processes).

This interpretation is consistent with previous experimental and theoretical studies, which demonstrated that G-quadruplex folding proceeds via a kinetic partitioning mechanism across a rugged free-energy landscape with multiple deep energy minima.^50–55^ These correspond to competing conformational basins and give rise to complex folding pathways populated by diverse intermediates (including G-triplexes, G-hairpins, and G-quadruplexes with shifted registers and of various molecularity^50–55^ ).

#### TBA-Gt

To test this interpretation, we acquired hyperpolarized spectra of a truncated construct derived from the Thrombin Binding Aptamer (TBA-Gt; Table 1 and Figure 5a).^56^ TBA-Gt folds into an intramolecular three-stranded structure, stabilized by Hoogsteen-type paired G.G.G triplets, representing an experimentally validated intramolecular model of a G-triplex-based G-quadruplex folding intermediate.^56,57^

**Figure 5:**
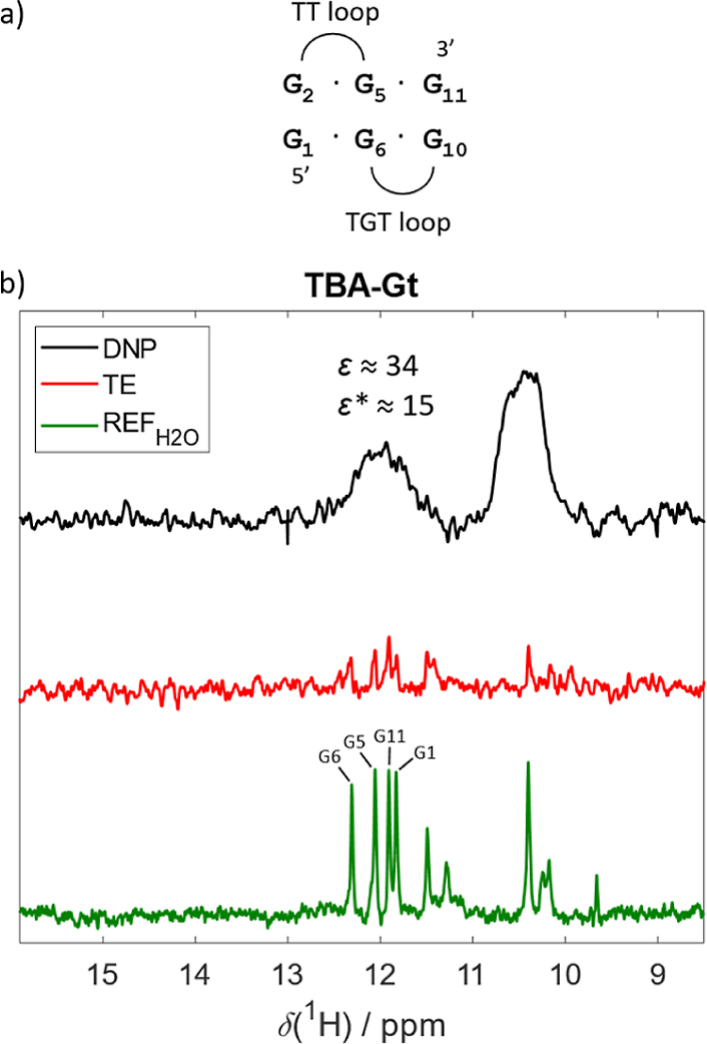
TBA G-triplex (TBA-Gt). (a) Schematic representation of TBA-Gt structure. (b) Comparison of the hyperpolarized spectrum of TBA-Gt (black), thermal equilibrium (red), and standard NMR reference (green). Enhancements are indicated for the most intense peak in the assigned region. The hyperpolarized spectrum was recorded with 3 scans, the TE with 4096 scans, and the REF_H_2_O_ with 16,384 scans. The resonance assignment was obtained from ref 56.

The hyperpolarized spectrum of TBA-Gt (Figure 5b) displayed two broad unresolved peaks centered at ∼10.4 ppm and ∼12.2 ppm. The spectral envelope of these peaks and their positions matched those observed in the REF_H_2_O_ spectrum. It should be pointed out that the HyperW-DNA mixing experiments were performed using a prototype device that eliminates the risk of microbubble formation, temperature and/or shim instabilities,^37,58^ which could otherwise account for the observed signal broadening.^18^ To further exclude this possibility, repeated measurements yielded identical outcomes (Supporting Information Figure S4). The observed signal broadening can be attributed to the intrinsic dynamic character of the TBA-Gt structure^56^ and conformational heterogeneity arising from chemical exchange. Most importantly, the spectral envelope and positions of these peaks closely resemble those observed in the hyperpolarized spectrum of c-myc-G4 (cf. Figure 4b). This finding supports the interpretation that the hyperpolarization-induced signals in the dDNP spectrum of c-myc-G4 reflect contributions from G-triplex-like folding intermediates, which remain undetectable in conventional NMR.

Two plausible scenarios may explain the observation of signals corresponding to folding intermediates in the dDNP spectra.

In the first instance, the rapid injection of hyperpolarized water during the dDNP process transiently perturbs the DNA folding equilibrium through subtle changes in ionic strength, temperature, or pH during mixing. These perturbations may be sufficient to partially unfold the G-quadruplex structure, thereby initiating a refolding trajectory that proceeds within the time scale of data acquisition in the dDNP experiments. In this case, the hyperpolarized signals act as reporters of this kinetic transition, and the observed enhancement results from an increased population of folding intermediates triggered by the mixing process.

In the second scenario, the perturbations associated with the mixing process are considered negligible. Here, the hyperpolarized signals arise from inherently present low-populated folding intermediates that are selectively enhanced. These signals benefit from the reduced stability of the folding intermediates, in combination with the HyperW effect, which preferentially boosts signals from species with lower stability (i.e., increased exchange dynamics).

#### T121–6-iM

To distinguish between the two above-mentioned scenarios, we acquired a dDNP spectrum of the T121–6-iM construct. It is a prototypical oligonucleotide model for the DNA i-motif; a tetra-stranded structure stabilized by intercalated C·C^+^H base pairs (Table 1 and Figure 6a).^59^ While the stability and structural characteristics of i-motifs differ from those of G-quadruplexes, DNA i-motifs are similarly known to fold via a kinetic partitioning mechanism.^60^ This involves multiple folding intermediates, i.e., partially folded species with incomplete base pairing and shifted C·C^+^H pairing registers.^60^ Thus, analogous to the situation observed for the c-myc-G4, the sparsely populated folding intermediates should coexist next to a fully folded T121–6-iM structure.

**Figure 6:**
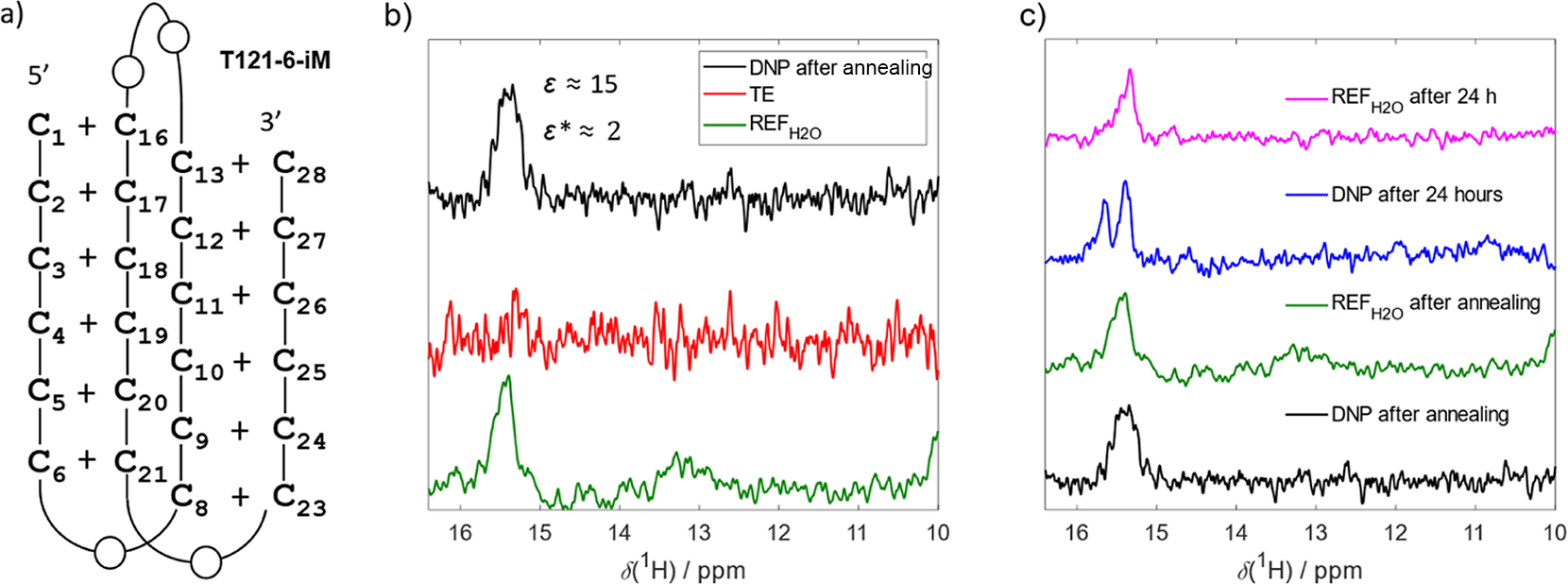
i-motif (a) Schematic representation of T121–6 i-motif (T121–6-iM) structure. (b) Comparison of the hyperpolarized spectrum of T121–6-iM (black), thermal equilibrium (red), and standard NMR reference (green) acquired immediately after the sample annealing. The hyperpolarized spectrum was recorded with 10 scans, the TE with 220 scans and the REF_H_2_O_ with 128 scans. (c) Annealing of T121–6-iM. The dDNP experiment was acquired 8 min after DNA annealing (black), and the dDNP experiment was performed 1 day after DNA annealing (blue). Reference experiments are shown in green and magenta. Note that the signal enhancement was only 20 directly after annealing (black spectrum), but reached >100-fold after 24 h (blue spectrum).

Still, a principal distinction between G-quadruplexes and i-motifs remains: the folding of i-motif constructs with long C-tracts (>5 cytosines) at neutral pH, such as T121–6-iM, occurs on a much slower time scale, typically minutes to hours for i-motifs, compared to seconds to minutes for G-quadruplexes.^60^ Thus, the slow folding kinetics of T121–6-iM provides a possibility to distinguish the two scenarios based on the time-resolved dDNP spectra via monitoring of the time-dependent changes in the populations of intermediates and folded species: According to above-mentioned scenario 1, the observed dDNP enhancements are due to increased population of folding intermediates triggered by the mixing process, inducing a shift in structural equilibrium from folded to misfolded species. In this case, the appearance of the dDNP spectrum should be largely independent of time following folding induction, as the mixing is expected to reset the equilibrium. In contrast, in scenario 2, the population of folding intermediates remains unaffected by the mixing process, and the populations of the intermediates are expected to redistribute gradually based on their thermodynamic stability, potentially leading to a distinct dDNP spectrum at different time points.

Consistent with previous reports, the conventional NMR spectrum (REF_H_2_O_) of T121–6-iM acquired immediately after sample annealing displayed a single, broad, asymmetric peak centered at 15.5 ppm, characteristic of C·C^+^H base pairing and indicative of a folded i-motif^3,59^ (Figure 6b). While the corresponding dDNP spectrum showed a single peak at nearly the same position, it was broader. As hypothesized for c-myc-G4, this broadening may be assigned to superimposed signals arising from an inherently heterogeneous dynamic ensemble of partially folded species representing the early phase of the folding process. Exploiting the extended times required to reach thermodynamic equilibrium between folded and partially folded species, we acquired REF_H_2_O_ and dDNP spectra of T121–6-iM 24 h after annealing (folding induction). Similar to the REF_H_2_O_ spectrum acquired immediately after annealing, the REF_H_2_O_ spectrum acquired 24 h postinduction featured a single peak at 15.5 ppm, corresponding to the folded species. In contrast, the corresponding dDNP spectrum after 24 h displayed not only the expected peak at ∼15.5 ppm, but also an additional broad, strong signal centered at ∼15.7 ppm (Figure 6c), likely corresponding to the kinetically trapped, partially C·C^+^H base-paired structures with shifted base pairing register coexisting with the fully folded T121–6-iM conformation.

Together, these observations support scenario 2 in which the hyperpolarized signals originate from low-populated folding intermediates that remain invisible to conventionally detected NMR and are selectively enhanced by HyperW/dDNP preferentially amplifies signals from less stable or more dynamically exchanging species, which expose their labile protons more frequently than the thermodynamically dominant, fully folded conformation.

#### LL3

Finally, to complement the results obtained for c-myc-G4 and T121–6-iM, we applied our HyperW approach to LL3 DNA (Table 1 and Figure 7a), which adopts an unusual tetra-stranded structure that features structural signatures of both G-quadruplex and i-motif.^61^ The LL3 fold is stabilized by two hemiprotonated C.CH^+^ base pairs (C7.C14 and C2.C19), forming a central core of the structure, capped on either side by a minor pseudo G/C/G/T tetrads assembled via a combination of the W–C type of base-pairing between G-C and G-T residues (Figure 7a).^61^ Notably and in contrast to the c-myc-G4 and T121–6-iM constructs, which fold into a G-quadruplex and an i-motif via a kinetic partitioning mechanism, this hybrid structure’s folding trajectory is expected to follow a “funnel”-like pathway, omitting semistable intermediates.^61^

**Figure 7:**
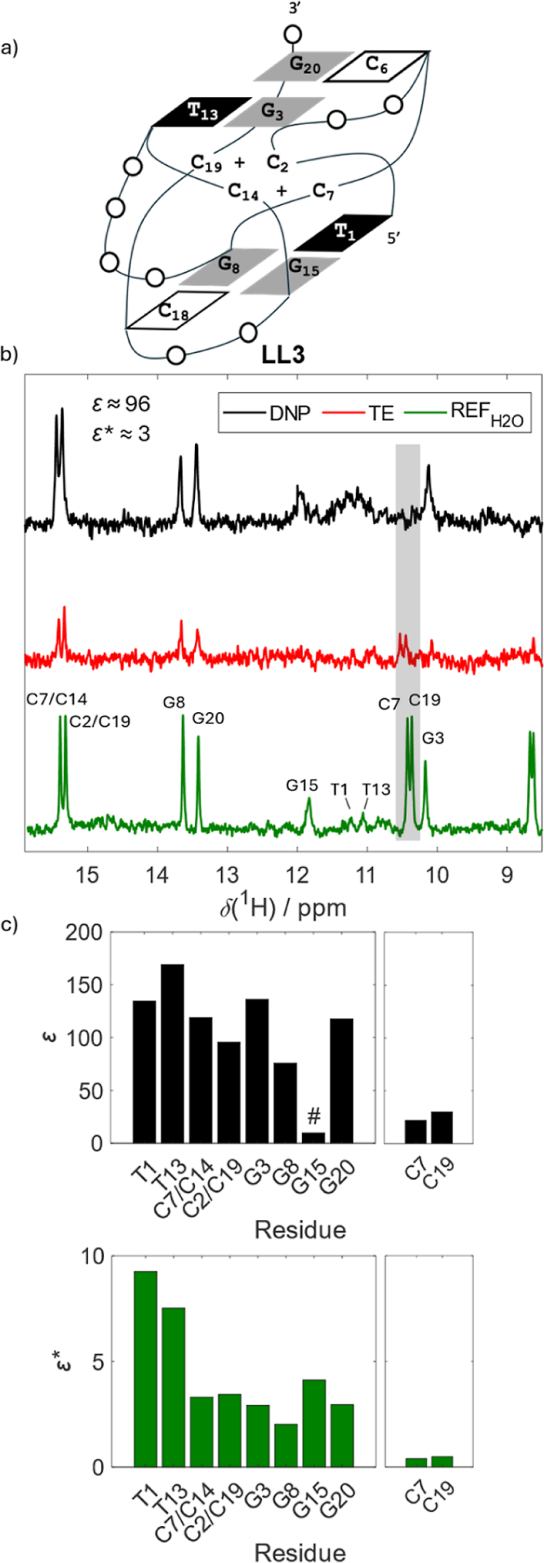
G-quadruplex/i-motif hybrid (LL3). (a) Schematic representation of LL3 structure. (b) comparison of the hyperpolarized spectrum of LL3 (black), thermal equilibrium (red), and standard NMR reference (green). Enhancements are indicated for the most intense peak. The hyperpolarized spectrum was recorded with 3 scans, the TE with 4096 scans, and the REF_H_2_O_ with 64 scans. (c) Residue-specific enhancement factors ε and ε*. Amino protons highlighted by a gray box in panel b are shown on the right-hand side. Residue G15, marked with the hash, showed no observable signal in TE; therefore, we report the SNR as a lower limit for the enhancement factor. The resonance assignment was obtained from ref 61.

In the dDNP spectrum of LL3 (Figure 7b), signals of hemiprotonated C·CH^+^ (C7·C14 and C2·C19) and Watson–Crick G–C (G8–C18 and C6–G20) bp closely matched those observed in the corresponding REF_H_2_O_ spectrum and exhibited moderate and uniform levels of enhancement (ε* ∼ 3; Figure 7c). A similar behavior was observed for the imino protons of G3 and G15, but not for those of T1 and T13, all involved in the G3·T13 and G15·T1 base pairs. In contrast to G3 and G15, the signals from T1 and T13 appeared markedly broadened in the dDNP spectrum, resulting in a single broad peak centered at their respective chemical shifts. This peak, consistent with the interpretation of dDNP data from other reported constructs, can be attributed to localized conformational heterogeneity, e.g., propeller-twisting-like motion,^62^ selectively facilitating imino proton chemical exchange from Ts and thereby the enhancement, relative to the G3 and G15 imino protons.

As such, the LL3 case also highlights the potential of dDNP for detecting dynamic regions in the DNA, even for moieties that remain mostly invisible with conventional NMR.

## Conclusions

We explored dDNP using hyperpolarized water as a robust and versatile approach to overcome longstanding sensitivity limitations in NMR spectroscopy of DNA and its polymorphs. Our results demonstrate an applicability across various canonical and noncanonical DNA motifs, including duplexes, triplexes, G-quadruplexes, i-motifs, and hybrid constructs, with signal enhancements of up to 370-fold. This sensitivity achieved by magnetization transfer from hyperpolarized water to imino and amino protons extends solution-state NMR to previously inaccessible sparsely populated DNA moieties and species, such as low-populated folding intermediates that remain invisible to conventional methods. dDNP, thus, offers insights into the kinetic partitioning mechanisms of G-quadruplex and i-motif folding, as well as local DNA dynamics. However, to fully exploit this potential, line broadening must be addressed in the future. Site-specific DNA labeling with nuclei such as ^19^F, ^15^N, or ^13^C could provide greater chemical shift dispersion, improving resolution and enabling identification of transient intermediates.

Looking ahead, the signal enhancement achieved by dDNP could open translational avenues in biomedicine, such as the noninvasive structural characterization of circulating DNA in biological fluids like blood plasma, where conformational states of cell-free DNA may report on disease-specific signatures, including cancer.^63^

## Experimental Section

### T121–6-iM

T121–6-iM was prepared in deuterated 50 mM potassium phosphate buffer with 100 mM KCl at pH = 6 and annealed at 90 °C for 10 min 150 μL of the sample were then pipetted into a 5 mm Shigemi NMR tube and inserted into the NMR magnet. dDNP acquisition started 8 min after the end of the annealing. The second part of the sample was left overnight at 22 °C and used to acquire a second dDNP experiment 1 day after the annealing. All dDNP measurements of T121–6-iM were performed at 17 °C.

### iTRP

iTRP was prepared as in ref 48. The sample was prepared in deuterated 30 mM potassium phosphate buffer, 300 mM KCl, 300 mM NaCl, 15 mM MgCl_2_ at pH 6. dDNP was performed at 30 °C.

### c-myc-G4

c-myc-G4 was prepared as in ref 49. DNA was dissolved in 50 mM potassium phosphate buffer with 100 mM KCl at pH = 6.

### DDD and LL3

Both were prepared in deuterated 20 mM sodium phosphate buffer with 200 mM NaCl at pH = 6 and annealed at 95 °C for 10 min. Samples were subsequently lyophilized and diluted by 150 μL of D_2_O before dDNP experiments, which were performed at 25 °C.

### TBA-Gt

TBA-Gt was prepared as in ref 57 . DNA was dissolved in 30 mM potassium phosphate buffer, 210 mM KCl, 0.2 mM EDTA, pH 7 and annealed at 95 °C for 10 min dDNP experiments were performed at 20 °C.

### Dissolution DNP

For DNP 200 mL of a solution of 15 mM TEMPOL in a mixture of 30% glycerol-d_8_ and 70% H_2_O was hyperpolarized at 1.4 K in a magnetic field of 6.7 T for 2500 s using continuous-wave microwave irradiation at 188 GHz. DNP samples were always freshly prepared to avoid ripening effects.^64^ A VDI microwave source was used together with a 16x frequency multiplier that provided an output power for the microwave of ca. 50 mW. The magnet-cryostat combination was purchased from Cryogenic Ltd. and operated as described in ref 58.

For the detection of the solid-state polarization, a 400 MHz Bruker NEO system was adapted to a ^1^H resonance frequency of 285.3 MHz by using a broadband preamplifier. The detection circuit and the external tune-and-match system were home-built, as described in refs 37 and 58 . To monitor the build-up, detection pulses with a flip angle of 1° were applied every 5 s.

After DNP, the sample was dissolved with a burst of 5 mL D_2_O at 1.5 MPa as described in ref 58. Resulting concentration of the hyperpolarized H_2_O was 2% v/v. The hyperpolarized liquid was then pushed with helium gas at 0.7 MPa to the detection spectrometer. The dissolution process employed a home-built pressure heater actuated with an Arduino microcontroller. A home-written Python-based user interface controls the dissolution and injection steps. The entire process, from the dissolution of the DNP mixture to the start of NMR acquisition, took about 2.5 s.

Detection was carried out using a 500 MHz Bruker NEO spectrometer equipped with a cryogenically cooled Prodigy BBO probe. The pulse sequence for detection corresponded to a series of single-pulse experiments (excite and detect). We used Gauss.1000 selective 60° covering a bandwidth of 5 ppm centered around a carrier frequency of 12.5 ppm. The recycling delay d1 was set to 1 s, and the additional acquisition time was 0.4 s.

The DNA solutions for dDNP contained 500 mM DNA before being 3-fold diluted with hyperpolarized water, as described in the main text. For the iM only, the concentration was 350 mM. Thus, the concentrations upon detection were ca. 166 and 116 mM, respectively.

Regarding the water concentration, after mixing with the DNA solution, the final HyperW concentration was 2% (v/v).

Enhancements were calculated as

$$\varepsilon =SNR\left(hyp\right)/SNR\left(TE\right)/{\left(NS\left(TE\right)/NS\left(hyp\right)\right)}^{1/2}$$

And

$$\varepsilon^{*}=SNR\left(hyp\right)/SNR\left({REF}_{H_{2}O}\right)/{\left(NS\left({REF}_{H_{2}O}\right)/NS\left(hyp\right)\right)}^{1/2}$$

Errors in ε and ε* were obtained by computing the average noise level σ as SNR uncertainty in combination with Gaussian error propagation.

### General Note on dDNP Sample Preparation

The individual samples were prepared as detailed above. The buffer concentrations in the dDNP experiments before 3-fold dilution with HyperW were 3× higher for compensation.

### NMR Spectroscopy

^1^H, and NOESY, experiments were recorded on a 500 MHz Bruker NEO spectrometer equipped with a cryogenically cooled Prodigy BBO probe. The 1D spectra with water suppression were recorded with the ‘zggpw5′ pulse program of the Bruker TopSpin 4 pulse sequence catalogue. The carrier frequency (o1p) was set to 4.7 ppm, and the spectral width (SW) was 24 ppm at a total acquisition time of 1.1 s ^1^H–^1^H NOESY was recorded using the “noesygpphw5” pulse sequence with 512 complex *t*_1_ increments of 170 μs each (O 1p = 4.7 ppm; SW = 9.8 ppm in both dimensions). All data were processed using TopSpin 4 and home-written scripts for MATLAB 2018b. Before the Fourier transform, all data were baseline corrected using fifth-order polynomials and apodized using either an exponential function (for 1D) or a 60° shifted squared sine bell function (for 2D).

For reference REF_H_2_O_ spectra, concentrations were 10–125 μM, depending on the previously reported conditions of the individual DNA targets.

## Supplementary Material



## Notes

Chemical shift assignments, NOESY data, repeatability data, residue-specific enhancements (PDF)
