# Supplementary material for: Hyperpolarized NMR Reveals Low-Populated Folding Intermediates in DNA
Source: J Am Chem Soc. 2025 Dec 8;147(50):46563–72. doi: 10.1021/jacs.5c17542 (PMC12715797; doi:10.1021/jacs.5c17542)
Supplement: Supplementary file 1 [file ja5c17542_si_001.pdf]

Hyperpolarized NMR Reveals Low-Populated Folding Intermediates in DNA

Milan Zachrdla[1], Ertan Turhan[1], Michala Bučková[2,3], Robert Hänsel-Hertsch[4], Lukáš Trantírek[2,], Dennis Kurzbach[1,*]*

[1]Institute of Biological Chemistry, Faculty of Chemistry, University of Vienna, Währinger Str. 38, 1090 Vienna, Austria.

[2] Central European Institute of Technology, Masaryk University, 625 00, Brno, Czech Republic.

[3] National Centre for Biomolecular Research, Faculty of Science, Masaryk University, Kamenice 5, 625 00 Brno, Czech Republic.

[4]Center for Molecular Medicine Cologne (CMMC), Faculty of Medicine and University Hospital Cologne, University of Cologne, 50931 Cologne, Germany.

**corresponding author: dennis.kurzbach@univie.ac.at; lukas.trantirek@ceitec.muni.cz*

- Supporting Information –

Table of Contents

Page S2………………………………………………………..Figure S1: LL3, DDD assignment

Page S3……………………..…………………………………..Figure S2: c-myc-G4 assignment

Page S4…………………………………………………………………..Supplementary NOESY

Page S5…………………………………………………………………..Figure S4: Repeatability

Page S6……………………………………….…………………….Tables with Enhancements

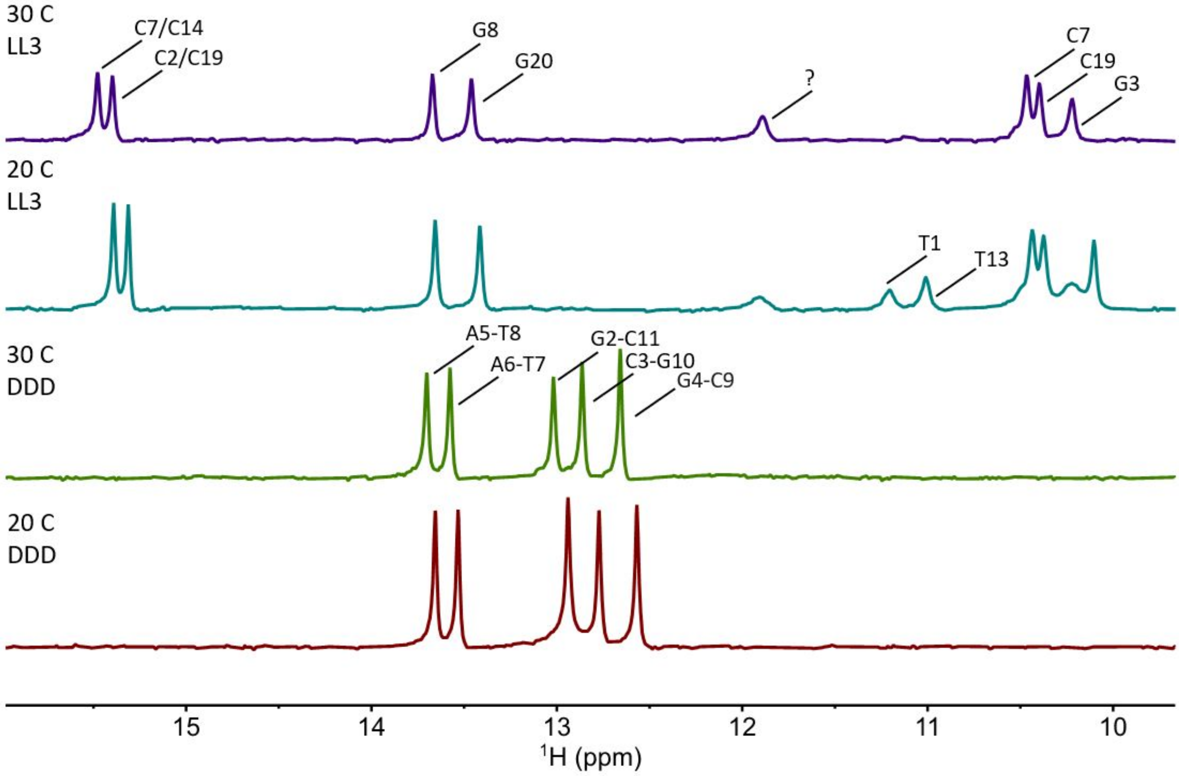

Figure S 1. Signal assignments for LL3 and DDD DNAs at different temperatures. The resonance assignment was obtained from ref. [1] (LL3) and [2] (DDD).

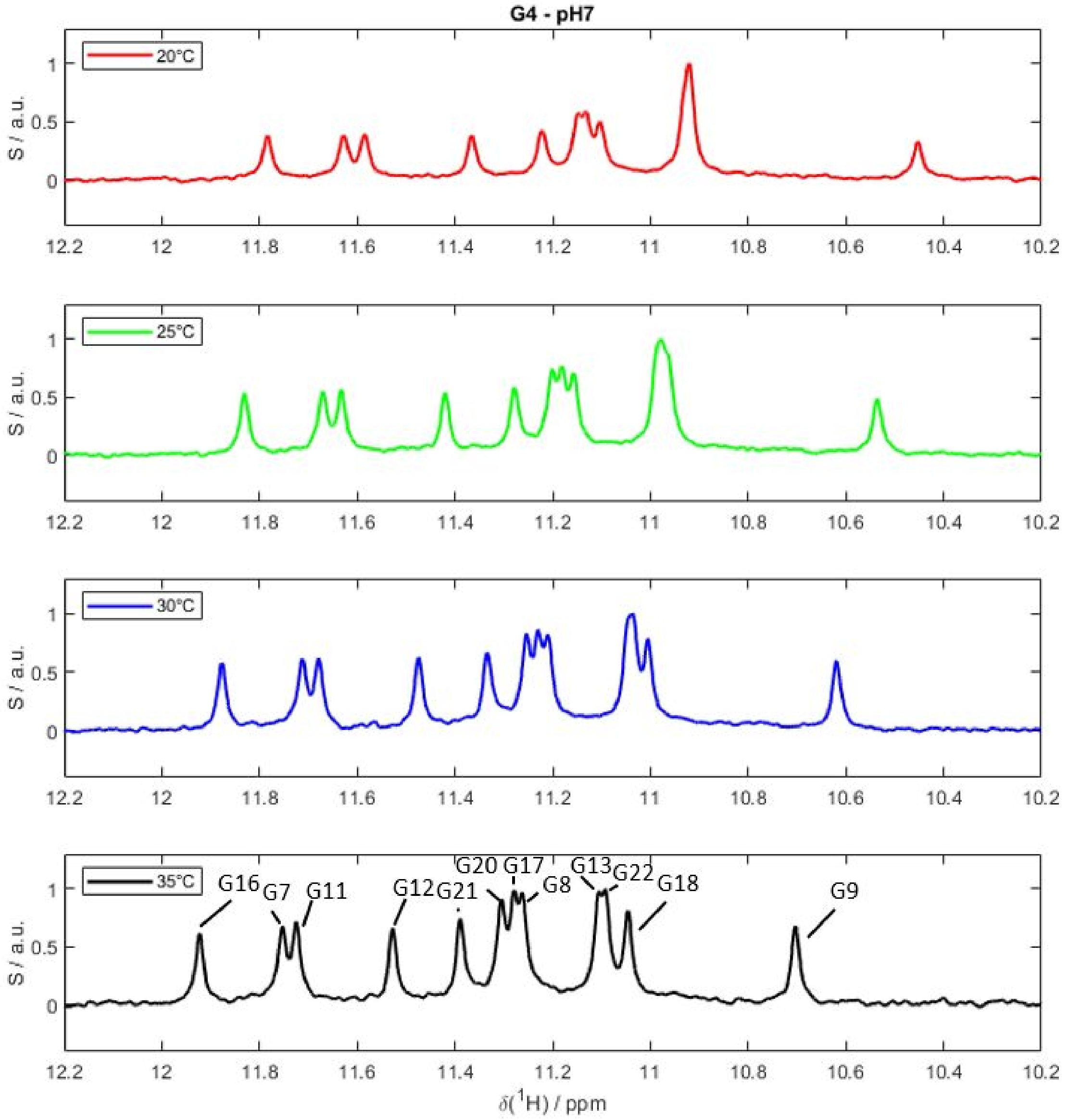

Figure S 1. Signal assignments for c-myc-G4 at different temperatures. The resonance assignment was obtained from ref. [3].

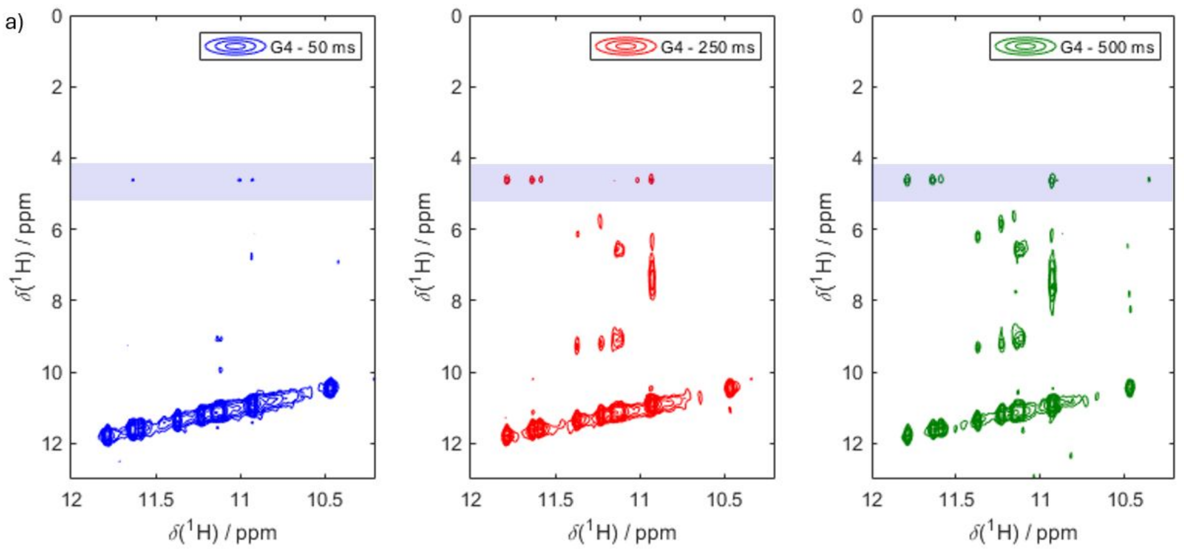

Figure S 2. Exemplary NOESY spectra of a c-myc-G4 at different mixing times. The slow appearance of the water exchange cross peaks is indicated by the grey bar.

Before diving deeper into the observed folding intermediates, we aimed to gain mechanistic insight into the polarization transfer process underlying dDNP-enhanced DNA spectra. We recorded conventional two-dimensional homonuclear NOESY spectra, representatively, of the used c-myc-G4 construct. Specifically, we focused on the exchange cross-peaks between water and imino protons at 4.7 ppm along the indirectly encoded dimension, which serve as direct reporters of hyperpolarization transfer pathways.

As shown in Fig. S3, strong water cross-peaks are observed for all imino resonances that exhibit high signal intensity in the hyperpolarized 1D spectra, confirming that magnetization transfer from hyperpolarized water occurs *via* proton exchange and subsequent spin diffusion. Only for relatively long mixing times >500 ms did we observe strong water-to-imino exchange peaks. This is clearly visible for c-myc-G4 in Fig. S3a. In other words, proton exchange is relatively inefficient compared, e.g., to exposed amides in IDPs. Thus, magnetization stemming from the surrounding water only transfers slowly to the imino moieties. Hence, while chemical exchange is slow compared to the solvent-exposed residues typically studied by HyperW, it remains effective nonetheless. Yet, clearly longer recovery delays between readout pulses are necessary for efficient signal enhancement and exchange of hyperpolarized protons between excitations. Thus, although it cannot be excluded that other DNA targets can be efficiently polarized with shorter recovery delays, >0.5 s should be chosen when aiming for a widely adoptable protocol, as representatively shown here for the c-myc-G4.

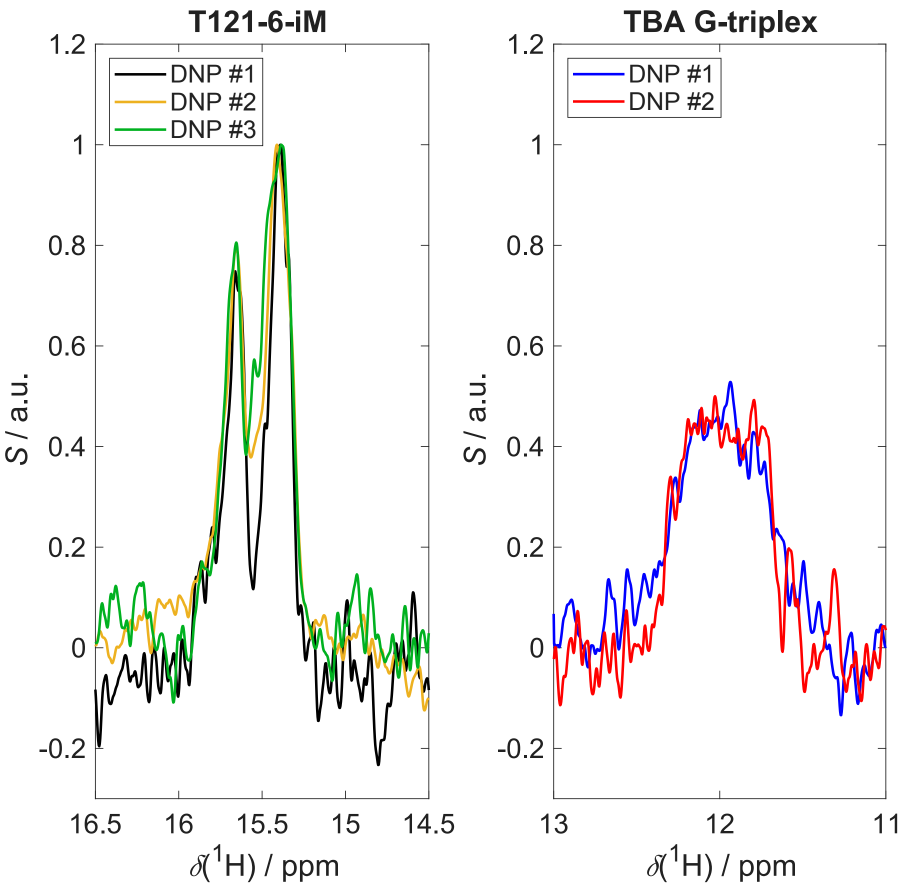

Figure S4. Line-shape repeatability of HyperW dDNP. Hybrid Sample Shuttling System allows for efficient mixing of HyperW with the DNA solution without affecting the line-shapes. Note that T121-6-iM measurement #2 has a higher SNR due to double the DNA concentration in order to evaluate the concentration dependence of the performed dDNP experiments.

Table S1. Residue-resolved enhancement factors.

DDD	ε	ε*
**T8**	42.459	1.803
**T7**	26.640	1.107
**G2**	129.009	6.698
**G10**	58.942	1.918
**G4**	30.156	1.112

iTRP	ε	ε*
**T12/ C+26**	201.485	49.870
**T13**	75.247	17.189
**T15,17**	71.508	16.604
**T23**	109.223	26.145
**T25**	102.671	20.149
**T27**	111.130	39.673
**G1**	86.985	33.523
**G3,5**	45.034	10.134
**C+24**	122.678	27.651
**C+24,26**	116.971	25.153
**C+26**	364.568	27.256
**C+24**	157.995	13.626

LL3	ε	ε*
**T1**	134.546	9.260
**T13**	169.021	7.527
**C7/C14**	118.833	3.316
**C2/C19**	95.689	3.447
**G3**	136.272	2.929
**G8**	75.869	2.026
**G15**	9.630	4.122
**G20**	117.715	2.952
**C7**	22.037	0.397
**C19**	30.031	0.496

References

1. Mir, B.; Serrano, I.; Buitrago, D.; Orozco, M.; Escaja, N.; Gonzalez, C., Prevalent Sequences in the Human Genome Can Form Mini i-Motif Structures at Physiological pH. *J Am Chem Soc* **2017,** *139* (40), 13985-13988.
2. Wu, Z.; Delaglio, F.; Tjandra, N.; Zhurkin, V. B.; Bax, A., Overall structure and sugar dynamics of a DNA dodecamer from homo- and heteronuclear dipolar couplings and 31P chemical shift anisotropy. *J Biomol NMR* **2003,** *26* (4), 297-315.
3. Ambrus, A.; Chen, D.; Dai, J.; Jones, R. A.; Yang, D., Solution structure of the biologically relevant G-quadruplex element in the human c-MYC promoter. Implications for G-quadruplex stabilization. *Biochemistry* **2005,** *44* (6), 2048-58.
